# Binocular rivalry under naturalistic geometry: Evidence from worlds simulated in virtual reality

**DOI:** 10.1093/pnasnexus/pgae054

**Published:** 2024-02-06

**Authors:** Shui'er Han, Randolph Blake, Celine Aubuchon, Duje Tadin

**Affiliations:** Center for Visual Science, University of Rochester, Rochester, NY 14642, USA; Department of Brain and Cognitive Sciences, University of Rochester, Rochester, NY 14642, USA; Institute for Infocomm Research Agency for Science, Technology and Research, Singapore 138632, Singapore; Centre for Frontier AI Research, Agency for Science, Technology and Research, Singapore 138632, Singapore; Department of Psychology, Vanderbilt University, Nashville, TN 37240, USA; Vanderbilt Vision Research Center, Vanderbilt University, Nashville, TN 37232, USA; Department of Cognitive Linguistic and Psychological Sciences, Brown University, Providence, RI 02912, USA; Center for Visual Science, University of Rochester, Rochester, NY 14642, USA; Department of Brain and Cognitive Sciences, University of Rochester, Rochester, NY 14642, USA; Department of Neuroscience, University of Rochester, Rochester, NY 14642, USA; Department of Ophthalmology, University of Rochester, Rochester, NY 14642, USA

**Keywords:** virtual reality, binocular rivalry, naturalistic visual experience, external validity, cognitive neuroscience

## Abstract

Binocular rivalry is a fascinating, widely studied visual phenomenon in which perception alternates between two competing images. This experience, however, is generally restricted to laboratory settings where two irreconcilable images are presented separately to the two eyes, an implausible geometry where two objects occupy the same physical location. Such laboratory experiences are in stark contrast to everyday visual behavior, where rivalry is almost never encountered, casting doubt on whether rivalry is relevant to our understanding of everyday binocular vision. To investigate the external validity of binocular rivalry, we manipulated the geometric plausibility of rival images using a naturalistic, cue-rich, 3D-corridor model created in virtual reality. Rival stimuli were presented in geometrically implausible, semi-plausible, or plausible layouts. Participants tracked rivalry fluctuations in each of these three layouts and for both static and moving rival stimuli. Results revealed significant and canonical binocular rivalry alternations regardless of geometrical plausibility and stimulus type. Rivalry occurred for layouts that mirrored the unnatural geometry used in laboratory studies and for layouts that mimicked real-world occlusion geometry. In a complementary 3D modeling analysis, we show that interocular conflict caused by geometrically plausible occlusion is a common outcome in a visual scene containing multiple objects. Together, our findings demonstrate that binocular rivalry can reliably occur for both geometrically implausible interocular conflicts and conflicts caused by a common form of naturalistic occlusion. Thus, key features of binocular rivalry are not simply laboratory artifacts but generalize to conditions that match the geometry of everyday binocular vision.

**Significance Statement**

Traditional perception science typically uses highly constrained, artificial laboratory conditions to elucidate the mechanisms underlying everyday perceptual behaviors. This has led to the development of phenomena that rarely occur in nature, raising questions about their ecological relevance. Virtual reality makes it possible to extend this “laboratory” world by creating scenes that mimic everyday environments. We show that binocular rivalry—a widely studied form of perceptual instability that occurs when the two eyes view incongruent images under artificial viewing conditions—also occurs under conditions that mimic natural occlusion geometry. Our study demonstrates the relevance of binocular rivalry for understanding everyday binocular vision and, more generally, the value of virtual reality technology as a means of bridging laboratory findings with natural vision.

## Introduction

Within the laboratory, studies of human visual perception generally deploy carefully crafted stimuli presented to observers under constrained conditions. For example, visual orientation perception is often studied with grating patches presented briefly under strictly enforced fixation. Although unnatural, these visual conditions have proved invaluable in revealing the fundamental properties of human vision (1–3). However, constrained and crafted stimuli can sometimes promote outcomes that depart from anything we typically experience during everyday conditions. One notable example is binocular rivalry (BR), the intriguing phenomenon revealed by optically presenting dissimilar monocular images to the corresponding retinal areas of the two eyes (4). This patently artificial viewing condition triggers a beguiling visual experience: rather than perceiving a stable, composite blend of the two images, one perceives an unstable, ongoing competition between the two images, with one dominating perception exclusively for a short period of time only to disappear from awareness as the other image achieves temporary perceptual dominance. Perception during these transition periods often resembles a dynamic amalgam comprising bits and pieces of each of the dissimilar images. Remarkably, these perceptual changes continue to cycle irregularly over time, despite the fact that this competition for phenomenological awareness is caused by *unvarying* physical stimulation (5).

Over the years, BR has emerged as a popular tool for studying perceptual bistability (6), predictive coding (7), neural concomitants of consciousness (8), and visual processing outside of awareness (9). BR has also been employed as a psychophysical tool for studying other aspects of human perception and cognition, including visual adaptation (10), eye dominance (11–13), mental imagery (14), spatial vision (15–17), multimodal sensory interactions (18–20), serial dependence (21), perceptual decision-making (22), and the effects of attention on visual plasticity (23, 24). BR has also been used as a proxy for assessing GABAergic inhibition in the human brain (25–29) and as an assay for examining neural dynamics in people diagnosed with psychiatric disorders (30).

However, despite BR's firmly established utility as a versatile research tool, it is unclear whether BR itself contributes to our understanding of everyday binocular vision and to what degree neural mechanisms engaged while experiencing BR reflect processes that could take place during natural behaviors. In other words, aside from being a useful inferential tool, is BR simply a laboratory artifact created using optical tricks (i.e. stereoscopic presentation) that force the two eyes to view different pictures? In this study, we consider two aspects of this question. First, do we encounter geometric stimulus conditions during everyday binocular viewing in which monocular images are in irreconcilable conflict between the two eyes? Second, does this interocular conflict give rise to BR alternations akin to those seen in the laboratory? The answer to the first question is definitely “yes.” The laws of perspective geometry dictate that binocular viewing of 3D scenes inevitably produces dissimilar stimulation of corresponding retinal areas (31). These conditions can and do arise in the following ways:

1. Objects located considerably farther or nearer than the plane of fixation form images on noncorresponding areas of the two eyes, creating large retinal disparities. Estimation of disparities encountered by observers in a variety of outdoor and indoor scenes shows that many naturally occurring retinal disparities exceed the limits of binocular fusion (32–34).
2. Binocular conflict also occurs when an observer purposefully uses one eye for visual tasks, such as taking a picture with a viewfinder camera, using a monocular microscope or a telescope, or sneaking a peak while hiding behind a tree. In these situations, the gray, amorphous cloud rendered by the closed eye's view conflicts with the scene viewed by the open eye. The outdoor scene dominates perception but can occasionally disappear within the closed eye's cloud (see González et al. (35), who refer to this effect as “weak BR”).
3. Occlusion can also lead to situations where one eye sees portions of the scene that are invisible to the other eye (36, 37). This can occur when a discontinuous surface near an observer occludes the visibility of portions of another, more distant surface, or when one side of an object is oriented more toward one eye than the other. One way to experience this conflict is to place a finger in front of one eye while fixating a word on a page; the occluded eye will see more of the finger, while the unoccluded eye will view more of the word on the page.

The answer to the second question—whether and how major interocular conflict in natural environments may lead to BR alternations—has been debated in the literature (38–40) but not resolved. This is because there are several key differences between naturally occurring interocular conflict and the conflict used to cause BR in the laboratory, including interocular differences in stimulus strength and task relevance, differences in attentional focus, and differences in viewing duration. Of particular relevance, there exists a fundamental difference in geometric plausibility between conventional laboratory BR and dissimilar monocular stimulation arising during natural viewing. Most laboratory studies of BR confront observers with conflicting interocular stimuli presented within context-sparse, ambiguous, and, of particular significance for the present work, implausible geometrical conditions. In contrast, interocular conflict associated with natural viewing situations arises within perceptual scenes replete with contextual information, including depth relations among objects. This key difference, to our knowledge, has not been systematically investigated despite strong evidence that vision and especially bistable perception are influenced by visual cues that support a coherent and meaningful interpretation (19, 41, 42). Evidence from prior studies is also mixed. Classical work reported that, at a very close distance, an object positioned to generate interocular conflict causes rivalry alternations (37). In contrast, low incidence of BR suppression was observed when rival stimuli were consistent with geometrically plausible physical occlusion (36). This earlier work, however, was conducted using typical, cue-impoverished laboratory conditions and did not fully test BR under geometrically plausible conditions. Still, it does suggest that the incidence of canonical BR alternations can depend on geometrical plausibility—a factor that has not been thoroughly investigated, presumably due to challenges in manipulating geometric relationships in the natural environment.

To address this gap in knowledge, we created a cue-rich 3D environment in virtual reality (VR) and examined how the ecological plausibility of geometrical cues affects the incidence and dynamics of BR. With VR, we can create naturalistic testing environments while also retaining the requisite degree of experimental control to carry out psychophysical studies. This includes controlling for attentional focus, stimulus strength,^1^ and task relevance. We used two different categories of BR stimuli, one portraying stationary form and the other portraying contours moving within an aperture. The impetus for this manipulation was both generalizability and the evidence suggesting that binocular interactions engaged by motion and by form arise within different pathways comprising the visual hierarchy (43, 44). The central hypothesis is that if ecologically plausible, geometrical cues signifying physical occlusion curtail the incidence of BR, we would expect this curtailment to generalize to both form and motion.

## Results

**Fig. 1.**
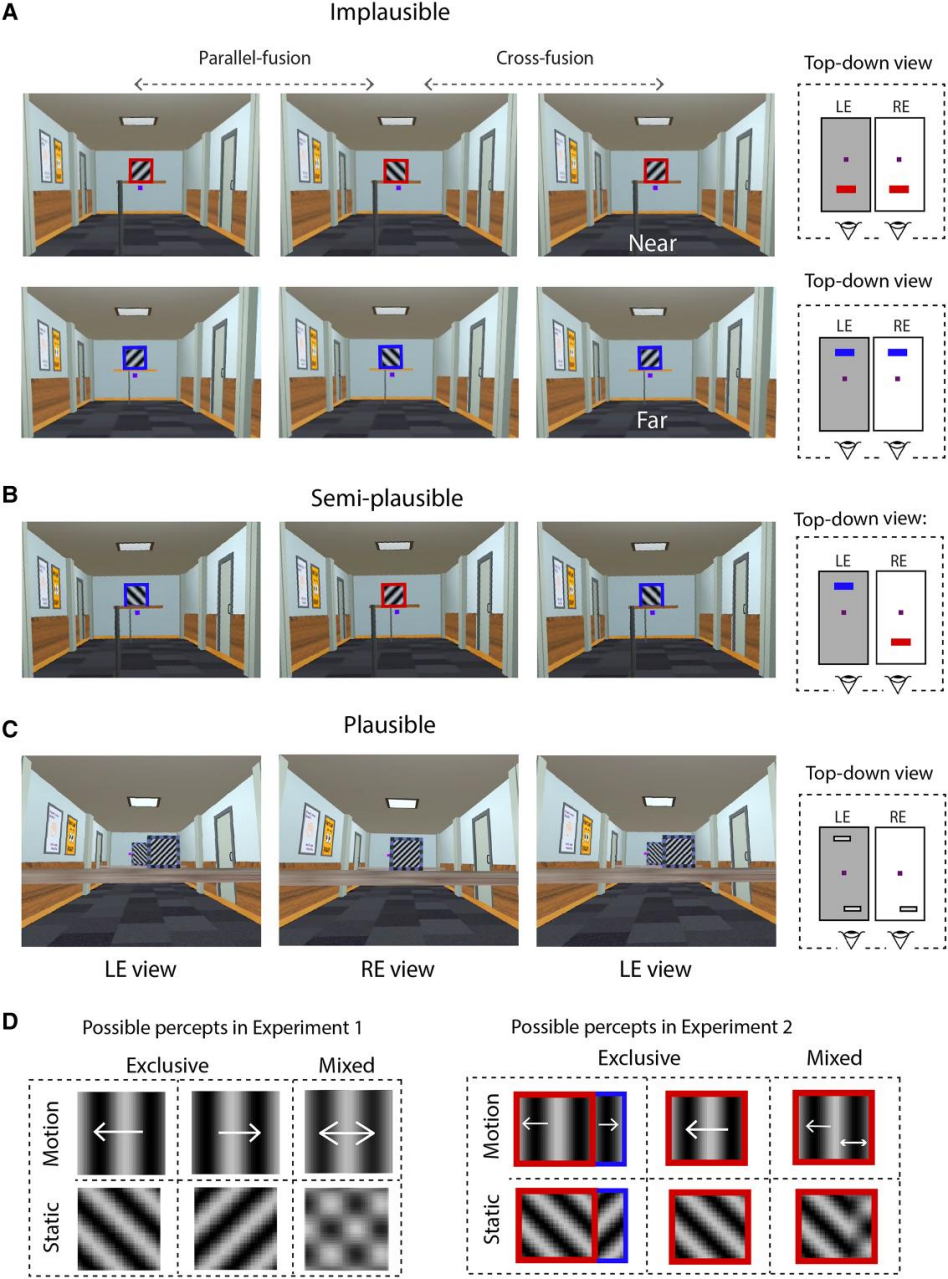
The spatial arrangements of stimuli in experiment 1 (panels A and B) and experiment 2 (panel C). All rival images were placed in a cue-rich 3D corridor, and the key manipulation was the configuration in which the rival images were presented. Specifically, the images were in the same depth plane (A, implausible, corresponding to the canonical BR configuration), separated in depth (B, semi-implausible), or spatially calibrated to simulate partial monocular occlusion (C, plausible). For illustrative purposes only, the far images and near images in experiment 1 are described by the respective top-down views. For each experiment, a top-down diagram of the different spatial layouts is also provided (right panels in A, B, and C). Small purple cubes/squares in images and diagrams indicate the fixation target. Three screen capture images are provided for each geometric condition, to allow readers to free-fuse and experience the depth manipulation. Readers who cross-fuse should fuse the left and center images (labeled LE view and RE view, respectively) and readers who use uncross-fusion should fuse the right and center images. Note that these screen captures contain image artifacts, such as barrel distortion artifacts. These artifacts were induced by the image acquisition process needed to separately capture left and right images and were not present in the experiment. D) Participants responded whenever they saw either one of two possible exclusive percepts, which corresponded to the left eye view or the right eye view. For experiment 1, this was a single motion direction or orientation. In experiment 2, exclusive percepts were perceiving either the occluding and partially occluded images with distinct orientations or motions, or just the occluding image with a single orientation or motion direction. Mixtures were defined as percepts that deviated from exclusive percepts, generally perceived as two superimposed orientations or motion directions.

The geometric plausibility of the interocular conflict was varied in a stepwise manner in two experiments (Fig. 1; Materials and methods). In experiment 1, we compared the effect of implausible and semi-plausible geometric conditions on BR alternation dynamics. The latter condition incorporated the depth separation between occluding and occluded objects but is considered semi-plausible because each eye can view only one rivaling grating (akin to situations described in point 3 in the Introduction). Experiment 2 used a plausible geometric condition that corresponds to partial monocular occlusion. Participants tracked their perceptual experience in both experiments.

### Experiment 1

**Fig. 2.**
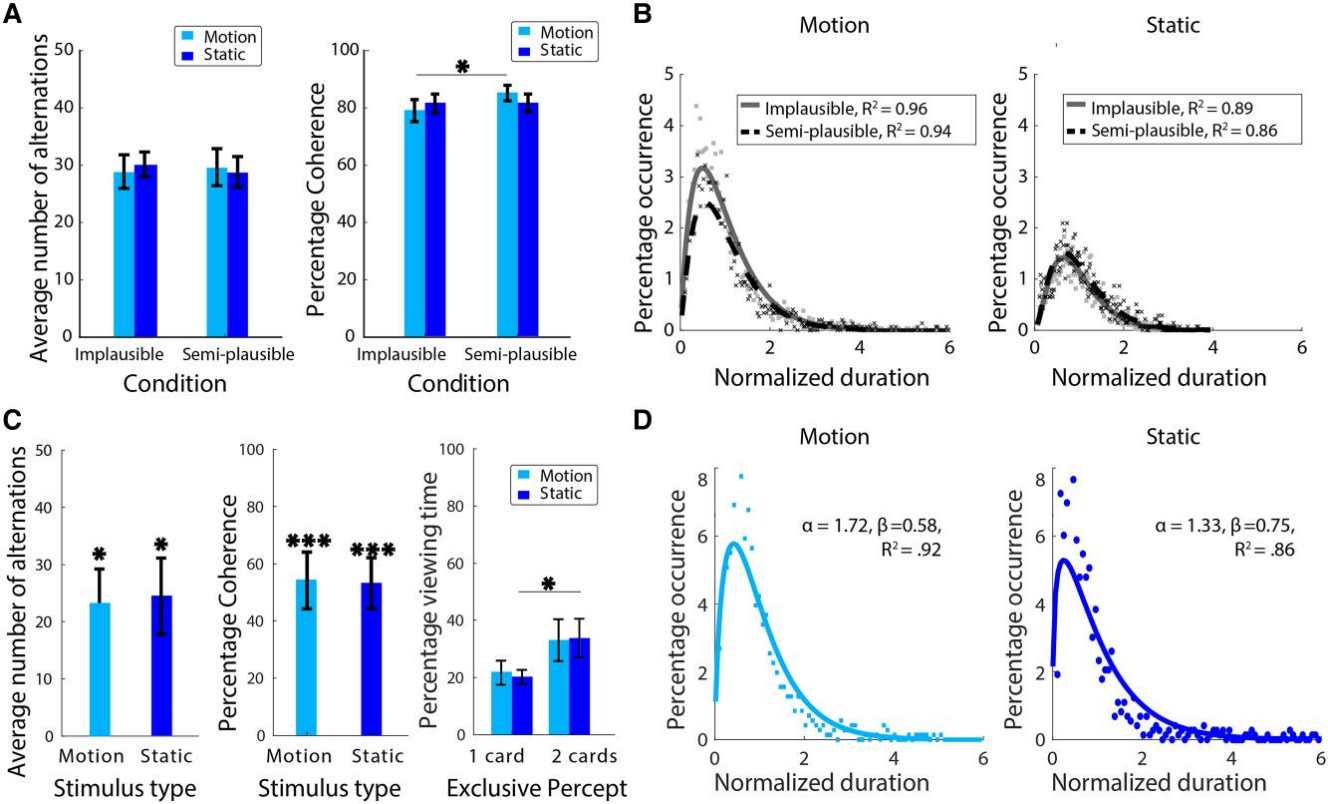
A) Experiment 1 results show that rivalry dynamics were largely unaffected by increasing geometrical plausibility. Regardless of stimulus type, comparable numbers of rivalry alternations were observed in the semi-plausible and implausible conditions (left panel). Rivalry alternations became more coherent when motion stimuli were separated in depth, a result that did not generalize to static stimuli (right panel). B) Distribution fits showed that the pooled dominance durations for both implausible and semi-plausible geometrical conditions were well-approximated by the gamma distribution. C) Similarly in experiment 2, presenting rivalry stimuli in a geometrically plausible layout did not eliminate BR incidence. Both the number of alternations and rivalry coherence remained considerably larger than zero (left and middle panels), although there is a noticeable decrease in alternations and rivalry coherence compared to experiment 1. This difference may be due to the larger stimulus sizes (45), which probably increased periods of mixed percepts and transparency in experiment 2. This is, of course, in addition to other small differences between the two experiments. We observed a significant bias toward seeing the nonoccluded view (i.e. two cards) compared with the occluded view (i.e. one card) when the data were split according to the type of exclusive percepts (right panel, see Fig. 1D for percept illustration). D) Similar to experiment 1, dominance durations in experiment 2 also followed the classic gamma distribution observed in laboratory rivalry studies, regardless of stimulus type. Asterisks represent statistical significance for parametric tests (*P < 0.05, **P < 0.01, ***P < 0.001), and error bars are standard errors.

We found that the number of alternations was comparable between the implausible and semi-plausible conditions (Fig. 2A). This was the case irrespective whether the rival stimuli were moving gratings (implausible mean: 28.9 alternations, SD = 10.92, semi-plausible mean: 29.7 alternations, SD = 12.11, *t*(13) = 0.72, *P* = 0.49; Bayes factors [BF] = 0.34) or static gratings (implausible mean: 30.2 alternations, SD = 8.0, semi-plausible mean: 28.84 alternations, SD = 10.0, *t*(13) = 1.29, *P* = 0.22; BF = 0.54). A similar result was observed with rivalry coherence when static gratings were presented (implausible mean: 81.6%, SD = 12.17, semi-plausible mean: 81.7%, SD = 11.63, *t*(13) = 0.53, *P* = 0.60; BF = 0.31). For moving gratings, however, the implausible condition (mean = 79.1%, SD = 14.32) had a lower proportion of exclusive dominance than the semi-plausible condition (mean = 85.1%, SD = 10.2, *t*(13) = 2.91, *P* = 0.01; BF = 4.82). In summary, we found that raising the geometrical plausibility of rival displays by introducing depth separation does not eliminate rivalry alternations. On the contrary, increasing geometric plausibility increased the proportion of exclusive rivalry for the motion stimuli.

Moreover, we found no evidence that increasing geometric plausibility affected the distribution of individual dominance durations (Fig. 2B). The normalized dominance durations for moving and static stimuli were generally well-described by a gamma function (*R*^2^ = 0.95, SD = 0.01 and 0.87, SD = 0.02, respectively). Results of the nonparametric permutation test showed that median normalized durations were significantly larger in the semi-plausible condition than the implausible condition for motion stimuli (0.82 vs. 0.77, *P*  *=* 0.02), but not for the static stimuli (0.92 vs. 0.88, *P* = 0.22) stimuli (Table S1).

### Experiment 2

Moving toward full geometric plausibility, experiment 2 tests rivalry dynamics under conditions of partial monocular occlusion. Because there was no natural comparison condition in experiment 2, the average number of alternations and rivalry coherence values were evaluated against a hypothetical mean of zero using two-tailed one-sample t tests. The results revealed a significant average number of alternations for moving gratings (23.2 alternations, SD = 17.15, *t*(7) = 3.82, *P* = 0.01; BF = 9.13) and static gratings (24.4 alternations, SD = 18.92, *t*(7) = 3.65, *P* = 0.01; BF = 7.68). Similarly, rivalry coherence was significantly higher than 0 for both moving gratings (54.8%, SD = 28.09, *t*(7) = 5.51, *P* < 0.001; BF = 45.2) or static gratings (53.8%, SD = 25.07, *t*(7) = 6.07, *P* < 0.001; BF = 72.2). In experiment 2, there are two different types of exclusive dominance precepts (Fig. 1D): the occluding frontal image and seeing both the occluding image and the distant, partially occluded image. Because the latter view is more informative and contains the complete 3D layout of the scene, we ascertained whether there was a bias toward either interpretation. We split the data according to the type of exclusive percept and computed the predominance values (i.e. the percentage of total viewing time for a percept). Participants tended to perceive the nonoccluded, two-image view (Fig. 1D) instead only perceiving the occluder (Fig. 2C, right panel), and two-tailed paired samples t tests showed that this trend reached statistical significance for static gratings (nonoccluded view: 33.8%, SD = 7.14, occluded view: 20.1%, SD = 19.12, *t*(7) = 2.71, *P* = 0.03; BF = 2.77) but not for moving gratings (nonoccluded view: 33.1%, SD = 20.61, occluded view: 21.7%, SD = 11.77, *t*(7) = 1.75, *P* = 0.12; BF = 0.99). Like experiment 1, the distributions of dominance periods for moving and static grating were well approximated by a gamma function (Fig. 2D and Table S1; *R*^2^_motion_ = 0.92, *R*^2^_static_ = 0.86).

### 3D simulation modeling analysis of interocular conflict

**Fig. 3.**
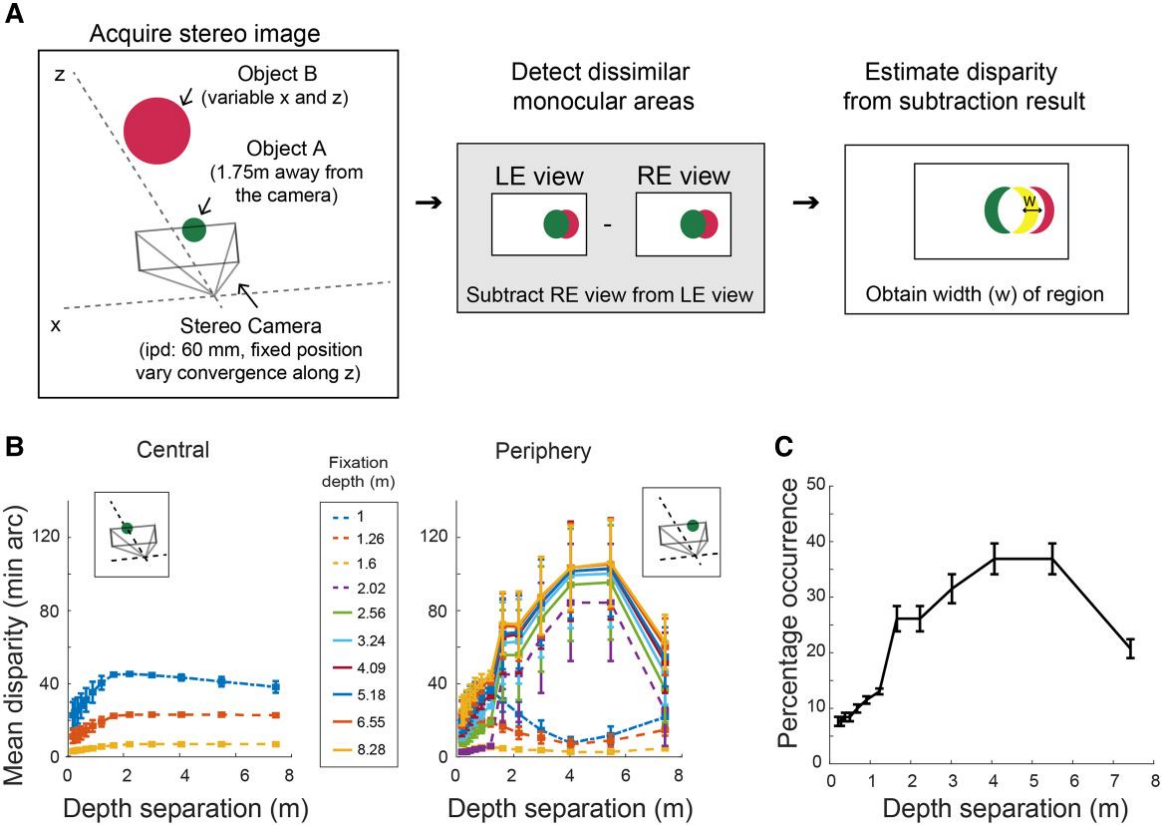
A) We estimated how often irreconcilable interocular conflict occurred between the eyes in an uncomplicated, sparse 3D scene. Two planar objects, each designated as the foreground or background (objects A and B, respectively), were viewed through a virtual stereo camera. Both objects were color coded and set to the same angular size. Object A was located at a fixed distance from the camera and was located 0^o^ (foveal or central presentation) or 4.9° (peripheral presentation) laterally from the midpoint of the camera, which represented central fixation. The distance from camera to fixation point was governed by the convergence angle of the stereo camera, which was varied to test different fixation points. The position of object B also varied laterally and in depth. Pairs of RGB stereo images were rendered for each of a variety of spatial layouts, and for each pair, the region of interocular conflict was detected by subtracting the left eye view image from the right eye view image. This yielded a database of output images which were used to estimate the disparity associated with the interocular conflict region (indicated by *w*). B) The relationship between depth separation and disparity magnitudes for foveal and peripheral presentations of object A. In sum, the disparity associated with the dissimilar region increased with depth separations, followed by a plateau or decrease at larger depth separations. Disparity also depended on the location of the object A (right panel), rising in magnitude when the occluding object is located away from the line of sight and further in depth from the fixation point. Note that there are only three fixation depths for foveal presentations as it is not possible for observers to fixate behind an occluding object. C) To estimate the incidence of irreconcilable interocular conflict, we estimated the frequency of computed disparities exceeding binocular fusional limits (46) and plotted percentage of these large, irreconcilable disparities obtained at each depth separation. Error bars in (B) and (C) are standard errors.

The results of experiments 1 and 2 show that naturalistic geometry does not curtail BR alternations. This prompted us to examine analytically the likelihood of interocular conflict caused by geometrically plausible occlusion in a simple visual scene containing two objects. To accomplish this, we simulated a simple 3D room that contained two planar objects positioned at different locations and at different relative depths (Fig. 3A). For each 3D object layout, a virtual stereo camera (63 mm simulated interpupillary distance) registered the left- and right-eye views of the layout. The camera settings were also varied to simulate different fixation distances. Using these left- and right-eye images, we estimated the amount of horizontal displacement (or disparity) associated with dissimilar monocular stimulation for each layout and fixation distance. Briefly, this analysis involves (i) detecting areas of dissimilar visual input generated by the two planar objects (Fig. 3A, middle panel) and (ii) computing the size of these regions (Fig. 3A, right panel; see also Supplementary Methods). Overall, we found that the horizontal displacement associated with interocular conflict increased with depth separations but plateaued and then eventually decreased at larger depth separations. The amount of conflict also depended on the fixation distance and positioning of the occluding object, rising in magnitude when the occluding object was displaced from the direct line of sight or located further behind or in front of the fixation point (Fig. 3B, right). Out of the total number of spatial arrangements tested at each depth separation, anywhere from 10 to 30% produced disparities exceeding the fusional limit estimates by Hampton and Kertesz (46) (Fig. 3C). This suggests that the occurrence of irreconcilable retinal disparities is not rare, and they exist as a consequence of partial occlusions and depth separations between objects (34). Note that our simulation results are obtained with a simple, uncluttered scene. Adding more objects will inevitably increase the incidence of dissimilar monocular stimulation and, possibly, large retinal disparities. A similar insight, incidentally, has been acknowledged in previous theoretical work (47) and dealt with explicitly using lateral inhibition among model neurons tuned to different disparities (48).

## Discussion

In the laboratory, BR alternations can be readily observed using optical means to achieve sustained dissimilar stimulation of the two eyes. Dissimilar monocular stimulation can also occur during natural viewing, but those situations rarely result in noticeable BR. Previous papers have pointed to opto-geometrical cues and/or interocular signal strength differences as factors contributing to the inconspicuousness of BR in the real world (36, 38, 39), and there is some empirical evidence supporting these claims (36, 49). In this study, we focused on isolating and testing the effect of naturalistic geometry on BR alternations. Naturalistic geometry is a key, yet understudied difference between naturalistic and laboratory-generated interocular conflict. We were inspired, in part, by earlier studies showing that in laboratory conditions, BR is affected by implied 3D surface properties such as simulated slant of ceiling and floor planes (50), surface texture coherence (51), and global context (52). Using VR technology, we implemented three different levels of geometrical plausibility in which observers were exposed to BR conditions, expecting that BR incidence to decrease with increasingly naturalistic geometry.

To our surprise, manipulating the geometrical plausibility of the 3D scene had essentially no impact on the incidence of BR alternations. In experiment 1, introducing depth separation in the semi-plausible condition did not affect the number of rivalry alternations (Fig. 2A, left panel). In fact, a significant *increase* in rivalry coherence was observed when motion stimuli were presented in a semi-plausible layout (Fig. 2A, right panel). Moreover, distributions of individual durations of exclusive dominance conformed to a gamma function (53), regardless of depth separation or stimulus type (Fig. 2B). Experiment 2 included a plausible, real-world condition: a stimulus arrangement rendered to portray partial monocular occlusion. Specifically, we paired a nearer stimulus viewed by one eye (i.e. the nonoccluded eye) with a partially occluded view of a more distant stimulus (36). With this layout, too, participants reported a high number of rivalry alternations that comprised a significant proportion of the total viewing time (Fig. 2C, left and middle panels). Lastly, individual dominance durations measured with motion stimuli and with static stimuli both were well-described by a gamma function, demonstrating again that naturalistic geometry had little effect on BR dynamics (Fig. 2B and D).

At first glance, the persistence of rivalry alternations evident in our results seems incompatible with the findings of Shimojo and Nakayama (36), who found relatively few instances where of BR alternations when dissimilar monocular stimulation was consistent with partial occlusion. Why might our results differ from theirs? We think the answer concerns stimulus differences, Shimojo and Nakayama (36) relied on a much smaller interocular conflict area (8–19 min arc vs. 2.5° in ours) and much shorter presentation durations (4 vs. 120 s). This latter factor is particularly notable because brief presentations impact the probability of observing switches as well as may be insufficient to reveal contextual effects in rivalry dynamics (54, 55). Indeed, when we tested observers in our experiment 2, but with rivalry stimuli 80% smaller and presented for 4 s, the reported incidence of state changes was quite low (Fig. S1), just as found by Shimojo and Nakayama (36).

By demonstrating that rivalry alternations can occur under naturalistic geometry, the current findings argue against the characterization of BR as a laboratory artifact. Why, then, do we not experience BR in our daily lives? Laboratory simulations of naturalistic geometry, such as ours and Shimojo and Nakayama (36), do not involve the differential depth of focus cues that occur under typical conditions in the natural world.^2^ While this was necessary to isolate the effects of geometry, it involves, to some degree, a cue conflict between occlusion cues and depth of focus cues, the effect of which is unclear. Nevertheless, some answers can be found in the existing BR literature. During natural viewing behavior, our fixations, on average, change several times a second (56), following a pattern and rate dependent on our current cognitive and perceptual state. The outcome is a series of changes in the visual input that create visual transients, which are known to disrupt BR (57, 58). We were not able to quantify the accuracy and stability of eye fixation in our study. However, we suspect the main effect of eye movements would be to reduce the incidence of BR alternations and thus work in the opposite direction of our main findings. We anticipate further developments in VR eye tracking will allow future research to carry out systematic assessments of eye movements under conditions of naturalistic interocular conflict.

A second plausible contributing factor to the lack of conspicuous BR alternations in the real world is attention. Observers in our study were instructed to attend and track BR alternations for an extended period. This is not typical of natural viewing behavior, where observers generally dynamically direct attention to objects of interest instead of tracking bistability over a sustained period. Studies show that the unattended competing signals do not engage BR processes (59, 60), but see Lee et al. (61). Instead, the unattended signals may take on a more stable format, resolving locally as a mixed percept (62) or as a combination of both signals (60). Lastly, even when (i) interocular conflict spans a large homogenous area, (ii) interocular conflict is viewed during extended visual fixation, and (iii) interocular conflict is salient enough to capture attention, perceptual selection may be biased toward the more ecologically useful alternative (63). This tendency is arguably evident in our experiment 2 results as well, where participants were biased toward perceiving the more informative, nonoccluded layout that contains the complete 3D layout of the scene (Fig. 2C, right panel). In the natural world, this bias toward the more useful interpretation may become enhanced by interocular blur differences (i.e. unfocused images of proximate occlusions and focused images near the point of fixation), which, as (38, 39) suggests, is demonstrative of BR's functional role in natural vision.

In summary, our findings support the external validity of BR as a tool for studying cognitive processes and natural vision. We found that canonical BR alternations persist over a full range of geometric plausibility, demonstrating that BR is not solely a laboratory artifact. While admittedly beyond the scope of the current study, future work could consider more aspects of naturalistic conditions, such as more realistic 3D objects and simulating differential depth of focus. In addition to the basic science that motivated the present work, this question also has applied significance. BR incidence in modern assistive visual devices and augmented reality technologies are potential avenues of investigation too, for there is some evidence that BR suppression may occur in head-mounted displays used in mobility (64). Our study also demonstrates the potential of VR and 3D technology in bridging the laboratory findings with natural vision, building on a growing and diverse set of studies using VR to study perception in naturalistic settings (65–68) and as a tool to assess and potentially rehabilitate binocular vision anomalies (69). We anticipate more fruitful pursuits taking advantage of VR and 3D technology as empirical tools. For example, one major question in the human binocular vision is the relationship between BR and stereopsis, the robust sense of depth perception derived from the relative, interocular positions of retinal images (70). Rivalry and stereopsis are thought to be separable, independent processes whose outputs are merged after completion (71). However, the latter is likely to take precedence (72) and seem to co-exist only with rivalry in separate spatial zones (73). Because these processes were previously studied under artificial laboratory conditions, future studies can consider studying them under more naturalistic conditions in VR or deploying 3D simulation models that might provide insights into the mechanisms that lead to the dominance of either process.

## Materials and methods

### Participants

Data were collected from 17 participants from the University of Rochester (age range: 18–32 years; 13 females, 4 males), including author C.A. Of the 17 participants, 9 females participated in experiment 1, and 3 females participated in experiment 2. Five participants performed both experiments (2 females, 3 males), leading to a sample size of 14 participants in experiment 1, and 8 in experiment 2. Sample sizes were determined using G*power (version 3.1.9.6). First, we entered the means and standard deviations provided in Dieter et al. (23) and found that a sample size of 4 produced an effect size of 2.16 (Cohen's *d*) with 98% power in a paired sample t test (alpha = 0.05, two-tailed). Next, we conducted additional sensitivity analyses using sample sizes for experiments 1 and 2. The results showed that the same t test was sensitive to an effect size of 0.81 with 80% power in experiment 1. For experiment 2, the two-tailed, one-sample t test was sensitive to an effect size of 1.16 with the same amount of power. We also confirmed that participants had normal or corrected-to-normal eyesight and normal stereovision as evaluated using the Titmus Stereo Fly Test. Experiments were approved by the Research Subjects Review Board of the University of Rochester and accorded with the Declaration of Helsinki. Participants provided written informed consent and were paid $15 for participation.

### Stimulus and apparatus

Figure 1 illustrates stimuli used in experiments 1 and 2. The cue-rich virtual environment—a 3D model developed in Blender 2.8.2—comprised a binocularly viewed, 11 m by 4 m corridor containing a textured floor, doors, and pictures hung on the walls. The 3D layout of these features of the corridor and the depth locations of the objects was portrayed using a combination of retinal disparity, linear perspective, and texture cues. The rival stimuli were either grounded to the floor with a binocularly viewed post (experiment 1; Fig. 1A and B) or presented on a binocularly viewed, horizontal platform that spanned the entire width of the corridor (experiment 2; Fig. 1C). Observers were instructed to fixate on a 3D cube (side = 0.88°) located in the center of the visual field, 6.5 m in virtual depth from the observer in experiment 1. Rival stimuli were 3.6° by 3.6° sinusoidal gratings, 0.75 cycles/degree in spatial frequency, and located ∼0.30° above the fixation cube. These gratings were either static or drifting horizontally (tested in different conditions). Static gratings had an orientation of 45° leftward in one eye and 45° rightward in the other eye. Drifting gratings were vertically oriented, with contours drifting leftwards at 6.7°/s in one eye and rightwards in the other eye. These rival gratings were presented in a geometrically implausible or semi-plausible layout in experiment 1. For the geometrically implausible layout (Fig. 1A), rivaling gratings were presented at the same virtual depth, either 3 m (Near) or 10 m (Far) in front of the participant. Note that this geometrically implausible layout is a direct VR analog to the dichoptic BR displays typically used in the laboratory. It is implausible because two 3D objects cannot simultaneously occupy the same location. To evaluate this particular geometrical constraint, we created a semi-plausible layout (Fig. 1B) that introduced a 7-m virtual depth separation between the gratings, which were located 3 or 10 m away from the participant. This spatial arrangement incorporates the depth separation between occluding and occluded objects. However, it is semi-plausible because each eye can view only one rivaling grating—a rare type of occlusion that occurs when the observer purposefully uses one eye (see point 3 in the Introduction).

In experiment 2, the rival stimuli were spatially arranged to simulate partial monocular occlusion under geometrically plausible conditions (Fig. 1C). Here, the Near grating was viewed by one eye, whereas the other eye viewed the Near grating and the partially occluded Far grating. To increase the occlusion area and maintain a comparable depth separation with the semi-plausible condition in experiment 1, the Near grating was brought closer to 0.7 m from the participant and the Far grating was located 8 m away, producing a depth separation of 7.3 m. The 3D fixation cube (side = 0.88°) was located at the right or left edge of the Far grating. The Near grating measured 7.6° by 7.6° and had a spatial frequency of 1 cycle/degree for the Static condition and 0.38 cycles/degree for the Motion condition. We kept temporal frequency comparable between experiments 1 and 2 (i.e. 5 vs. 4 Hz, respectively), in consideration of its effects on rivalry alternations (74). This produced a resultant speed of 10.5°/s for the Near moving grating. The Far grating was subjectively adjusted to match the front grating in size and spatial frequency during a pilot phase. The stimulus alignment is particularly important for experiment 2 which is why participants in this experiment viewed the virtual stimuli, while their head positions were stabilized by a chin rest. This additional maneuver helped maintain a fixed degree of interocular conflict in experiment 2.

All stimulus presentations were developed in Unity 2019 (Unity Technologies) and displayed using a head-mounted device (Oculus Quest 2). Note that the main purpose of VR here is to allow easy manipulation of 3D geometry and provide an immersive *visual* experience. Participants did not move in the 3D virtual space, as is sometimes done in other VR studies. The non-/semiplausible layouts were presented in a randomized order in experiment 1, and the plausible condition was tested separately in experiment 2. In addition, the orientation or motion content of the rivaling images was balanced between the eyes in experiment 1. In experiment 2, the position of the frontal image was randomized, such that it would occlude the right eye on 50% of the trials and the left eye on the remaining trials.

### Procedure

The main task in both experiments was for participants to indicate periods of exclusive BR dominance of one stimulus by squeezing and holding the left trigger and periods of exclusive dominance of the other stimulus using the right trigger. Participants reported exclusive dominance when they saw a grating with a distinct orientation or motion direction in experiment 1, or when they saw the frontal grating with a single orientation or motion direction in experiment 2 (Fig. 1D). Participants squeezed neither trigger when experiencing mixed dominance (i.e. portions of both orientations or both motion directions were perceived simultaneously; Fig. 1D). Both experiments recorded dominance tracking for a period of 2 min per trial. Motion and static stimuli were tested in separate, counterbalanced blocks, within which two trials per depth condition were presented. Prior to data acquisition, participants were administered a practice session to become familiar with task requirements. In experiment 2, an additional calibration phase was performed before BR dominance tracking. In this phase, participants were presented with, for example, a right-eye monocular view of the 3D scene and were instructed to shift the far grating until it was exactly and completely occluded by the near grating. This created geometrically plausible interocular conflict when both eyes were open, in which the right eye viewed only the occluding grating and the left eye viewed both the occluding grating and the left edge of the occluded grating.

### Analyses

Trials corresponding to a specific experimental condition (e.g. Static, Near condition) were pooled for each participant. This yielded 8 min of tracking data per condition, except for two participants in experiment 1 who completed 4 min of tracking for each static or motion condition. Trigger responses shorter than 300 ms were deemed finger errors (75) and were removed from the dataset. In some instances, participants may perceive the same exclusive orientation or motion direction on successive periods of exclusive dominance, separated by a period of mixed dominance. Known as return transitions (23), these were combined into the same dominance period if the two trigger responses were separated by <300 ms.

Using the remaining dataset, we quantified rivalry dynamics by computing (i) the average number of perceptual alternations per trial and (ii) the percentage of total viewing time that participants reported viewing an exclusive percept, an index we term rivalry coherence. These two metrics summarize the incidence of BR and were assessed using parametric analyses. BFs were also computed in our analyses, and these represented the extent to which the alternative hypothesis is favored over the null hypothesis. Because the implausible condition of experiment 1 included two distance conditions (near and far; Fig. 1A), we first determined whether the presentation depth plane affected rivalry dynamics. For motion stimuli, neither the alternation rate (Near: 29.7 alternations, SD = 10.50, Far: 28.1 alternations, SD = 12.22, *t*(13) = 0.94, *P* = 0.36; BF = 0.39) nor the rivalry coherence (Near: 80.2%, SD = 15.0, Far: 77.9%, SD = 14.88, *t*(13) = 1.02, *P* = 0.33; BF = 0.42) differed significantly between the Near and Far depth planes. Presentation depth plane also did not significantly affect the rivalry coherence for static stimuli (Near: 81.3%, SD = 13.28, Far: 81.8%, SD = 11.3, *t*(13) = 0.53, *P* = 0.60; BF = 0.31), but there was a small increase in alternations when the stimuli were placed in the farther depth plane (Near: 28.8 alternations, SD = 6.93, Far: 31.6 alternations, SD = 9.55, *t*(13) = 2.22, *P* = 0.045; BF = 1.71). We then collapsed the data over the two depth planes portraying implausible layouts and compared those data to the semi-plausible condition—the main comparison of interest.

In addition, we estimated the overall distributions of dominance durations for each geometrical condition (i.e. implausible, semi-plausible, and plausible geometry). Individual datasets were first screened to exclude responses truncated by the end of the trial and outlier durations larger than 2.5 times the median absolute deviation. This procedure removed 7.1% (SD = 5%) of data points from each individual dataset. The remaining data points were pooled together for each geometrical condition, normalized to the condition mean, and binned into 100 ms epochs. Because histograms of BR dominance durations tend to be asymmetrically skewed toward longer durations (76), the binned data were fitted with a gamma probability distribution function using a maximum likelihood estimation procedure. Permutation tests were used to compare the median normalized durations between implausible and semi-plausible conditions in experiment 1 (this analysis was not conducted in experiment 2 as there was no natural comparison condition). In the permutation tests, the difference in medians (test statistic) was computed for each stimulus type, using the datasets in the semi-plausible and implausible conditions. An empirical distribution of median differences was then compiled from 1,000 permutations of the implausible and semi-plausible datasets. The *P*-value was the proportion of permutated statistics larger than the test statistic.

## Supplementary Material

pgae054_Supplementary_Data

## Data Availability

The data and analysis codes for this study are available at the Open Science Framework (URL: https://osf.io/r5w7f/?view_only=b427ce68d25342bdbcf67ccab5210ac5).

## Footnotes

1 In natural environments, objects located away from fixation (both close and far) tend to project a blurrier image than closer, conflicting image sources. This creates a confound, where the absence of BR in the natural world may be explained by the naturalistic geometry and/or differences in optical image clarity. An exception occurs when an observer with a naturally small pupil observes a sunny outdoor scene. In this scenario, the change in diopter power for our stimulus distances is within the depth of focus of the eye (i.e. 0.18D toward 3 m and 0.05D toward 10 m in experiment 1) and would produce comparable image clarity for close and distant sources. Thus, to some extent, our VR setup involves a cue conflict between geometric cues indicating partial occlusion and a lack of differential depth of focus cues. Nevertheless, it enables us to isolate the effects of geometry while mimicking conditions relevant to natural outdoor viewing with a small pupil.

2 See footnote 1.
