# Supplementary material for: Binocular rivalry under naturalistic geometry: Evidence from worlds simulated in virtual reality
Source: PNAS Nexus. 2024 Feb 6;3(2):pgae054. doi: 10.1093/pnasnexus/pgae054 (PMC10877069; doi:10.1093/pnasnexus/pgae054)
Supplement: pgae054_Supplementary_Data [file pgae054_supplementary_data.docx]

**Supplementary Methods**

*3D model analysis of interocular conflict.* The simulation model was developed and conducted using the open-source modelling software tool Blender 2.8.2. Two planar objects (each with radius = 1.64^o^) were used to represent foreground and background surfaces (Figure 3A). These were placed in a 3D virtual scene and viewed through a stereo camera with an interocular distance of 60 mm and a focal length of 50 mm. The stereo camera was positioned at the origin, and the foreground object was located 1.75 m away from the camera. The foreground object was horizontally aligned with the camera (foveal or central presentation) or shifted 4.9^o^ from the centre (peripheral presentation), which is synonymous with the point of fixation. The position of the background object was varied with respect to the foreground object. Specifically, the depth separation between the two objects was one of 13 values (i.e., 0.2, 0.27, 0.37, 0.67, 0.9, 1.22, 1.65, 2.22, 3.01, 4.06, 5.49 and 7.42 metres), and the centre of the background object was one of 10 displacement values (i.e., 0.1, 0.13, 0.17, 0.2, 0.23, 0.27, 0.3, 0.33, 0.37 to 0.4 metres) to the right of the foreground object. Fixation distances were varied by changing the location of the camera’s projection plane, and ten fixation distances were included in the simulation (i.e., 1, 1.26, 1.6, 2.02, 2.56, 3.24, 4.09, 5.18, 6.55 and 8.28 metres away from the camera). Because it is not possible to fixate centrally behind a central occluding object, images were only rendered for the 1, 1.26 and 1.6 m fixation points when the foreground object was in a central position. To simplify the detection of conflict regions, the objects were colour coded (i.e., green or red) and a pinhole camera model was used to render focused, RGB stereo images. Areas of dissimilar input were detected by subtracting the red, green and blue channel values of the left stereo image from the right stereo image.

From these procedures, a total of 1690 pairs of stereo images were generated. We calculated the absolute difference between the left and right eye images for each pair of stereo images. This operation produced an output image that contained yellow pixels at regions of interocular conflict, because the foreground and background objects were coded green and red respectively (Figure 3A). The horizontal displacement (or disparity) associated with the region of interocular conflict was estimated with its maximum width in visual angle. We then computed the average disparity at each depth separation and fixation depth, collapsing the data across the different lateral displacements. Data corresponding to central and peripheral presentations of the foreground object were analyzed and plotted separately in Figure 3B.

**Table S1.** Gamma distribution parameters in Experiments 1 and 2

| Experiment 1: Motion stimuli | | | | | | |  |  |
| --- | --- | --- | --- | --- | --- | --- | --- | --- |
| Geometrical condition | | | Shape (α) | | Rate (β) | | | R^2^ |
| Implausible | | | 1.99 | | 0.49 | | | 0.95 |
| Semi-plausible | | | 2.17 | | 0.47 | | | 0.94 |
| Near, Implausible | | | 2.17 | | 0.45 | | | 0.94 |
| Far, Implausible | | | 1.84 | | 0.54 | | | 0.92 |
|  |  |  | |  | |  |  |  |

Experiment 1: Static stimuli

| Geometrical condition | Shape (α) | Rate (β) | R^2^ |
| --- | --- | --- | --- |
| Implausible | 2.92 | 0.34 | 0.89 |
| Semi-plausible | 2.73 | 0.38 | 0.86 |
| Near, Implausible | 2.94 | 0.35 | 0.82 |
| Far, Implausible | 2.91 | 0.33 | 0.85 |

Experiment 2: Plausible condition

| Stimulus type | Shape (α) | Rate (β) | R^2^ |
| --- | --- | --- | --- |
| Motion | 1.72 | 0.58 | 0.92 |
| Static | 1.33 | 0.75 | 0.86 |

**
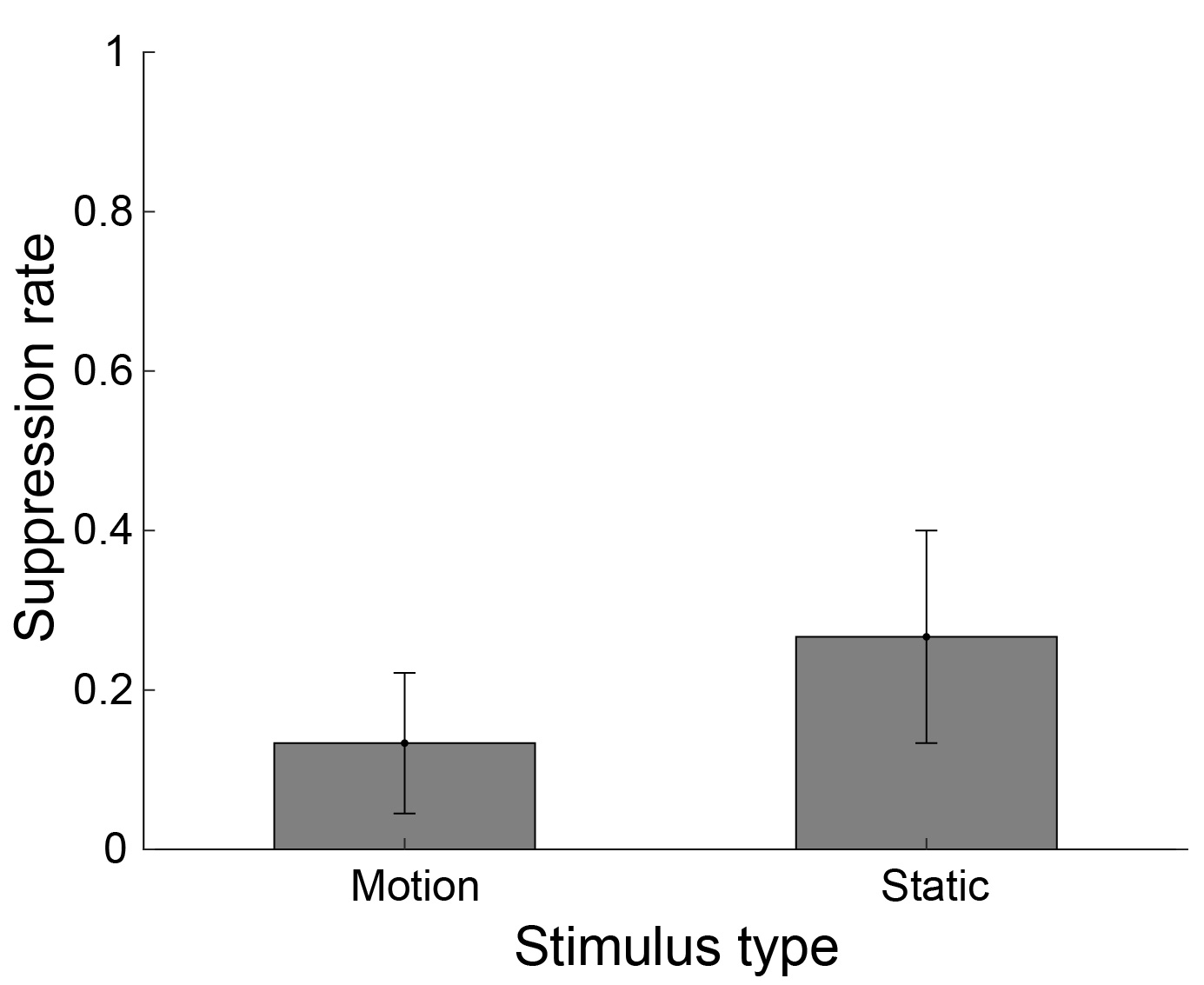
**

**Figure S1.** To better understand the discrepancy between Experiment 2’s results and the observations made in Shimojo and Nakayama (1990), we made the rivalry stimuli 80% smaller (~1.6º as compared to 7.6 º) and used a considerably shorter viewing duration (4s). We piloted these test conditions on three participants (author SH and two naive participants), who completed 10 trials for each stimulus type. Participants also completed a practice phase to ensure that they understood the task requirements. Mirroring Shimojo and Nakayama (1990), trials where participants experienced rivalry alternations or mixed percepts were considered BR suppression trials, and we computed the proportion of these trials to obtain the suppression rate. The results revealed suppression on only ~20% of trials, which is similar to what Shimojo and Nakayama (1990) found in their study. Altogether, this indicates that the relative absence of rivalry alternations in Shimojo and Nakayama (1990) for their geometrically plausible condition may be related to the stimulus properties used in their study.
